# 

**Published:** 1819-04-01

**Authors:** 


### CORRESPONDENCE.

Dr. Dickson's valuable Paper on Phlegmasia Dolens; Dr. Wight's important Essay on the Treatment of Fever, founded on the basis of pathological anatomy; Dr. Jeffray's Case and Dissection of Hydrocephalus; and Dr. Mathias's Case of Hernia with Hæmorrhage, have been unavoidably postponed.

We regret that Dr. Davis's report of Infantile Diseases, treated at the Universal Dispensary for Children, came too late for insertion, as the last sheet is always put to press on the 16th day of the month preceding publication, in order that the Journal may be ready to send off to Edinburgh and Dublin by the 25th. We shall be happy to give the *half year's* report in our next.

Dr. Carbutt's valuable paper is received.

---

We have been repeatedly solicited to mark the Prices of Works. This we dare not do, without subjecting ourselves to the advertisement duty. In the country, our readers may form a tolerably good estimate of the price of Octavos, by calculating each work at three shillings for every 100 pages, and if there be plates, counting them at sixpence each. Thus Dr. Armstrong's work on Typhus runs 340 pages, and sells for 10s. 6d. which illustrates the foregoing rule.

The complete List of Subscribers is unavoidably deferred till our next Volume.

*Nos.* 1, 2, *and* 3, *of this Journal are reprinting with all possible expedition, and will be ready for delivery by the* 1*st of July.*

*** In this and every succeeding Number of the Journal will be found 100 pages of *concentrated* Analytical Review, and 60 pages, of Original and Miscellaneous Matters.

☞ The next Number will be published on the 1st of July.

### Additional Subscribers.

Alexander, Mr. John, Surgeon, Newbury, Berkshire
Ange, Mr. James, Surgeon, Fareham, Hants
Anderson, Mr. William, (2) Surgeon, H. M.S. Grasshopper
Bailey, Dr. Reading, Berkshire
Barry and Co. Broad Street Buildings
Bent, Mr. Diss
Bullen, Mr. Surgeon, Ipswich
Bullen, Mr. George, Surgeon,
Burrows, Dr. Gower Street
Campbell, Dr. Dougal, Picardy Place, Edinburgh
Clarke, J. F. Esq. Surgeon, Currie
Corsan, John, Esq. Royal Navy
Cosgrave, Mr. Batavia
Davies, Dr.
Dobson, Dr. Chatham Division of Marines
Fraser, Rev. Simon, Edinburgh
Freer, John, Esq. York Hospital
Fox, Mr. Surgeon, Huntingdon
Gordon, Alexander, Dr. No. 2, Finsbury Square, London
Graham, Dr. William, late of Jamaica
Hadon, Mr. Sloane Street
Halkyard, Edward, Esq. Oldham, Lancashire
Hamilton, Dr. Finsbury Square, London
Hillyar, Dr. Royal Navy
Hughes, Dr. Physician to the Forces, Barbadoes
Jones, Mr. Surgeon, Princes Street, Cavendish Square
Jordan, Mr. St. James's Square, Manchester
Kedgell, Mr. Richard, Surgeon, Wellington, Somerset
Lyster, Mr. 7th Dragoon Guards
May, S. Esq. Surgeon Ilfracombe
Metcalfe, Thomas Thomson, Sheffield
Montgomery, Mr. A. Surgeon, Newman Street
Moore, Mr. Surgeon, Woodbridge
Mullins, Mr. John
Murray, Mr. John, Edinburgh
Newsom, Mr. Surgeon, Rochester
Nunn, Mr. Joseph, Surgeon, Holbrook
Nussy, Mr. St. James's Street
O'Malley, James, Esq. Surgeon of the 11th Light Dragoons, Bengal
Parkinson, Mr. George, Racquet Court, Fleet Street
Perkins, Mr. Measham
Prevost, Dr. Switzerland
Price, Dr. Wm. United States
Price, Mr. William, Surgeon, R. N.
Pyne, Mr. William Collard, Surgeon, Wellington, Somerset
Rodmel, John, Esq. Surgeon R. N.
Russell, Mr. George Ireland, London
Ryland, Mr.
Sainsbury, W. M. D. Corsham, Wilts
Sherwood, Ralph, Esq.
Shoolbred, Mr. John G. Charles Town, South Carolina
Stacy, Mr. London Hospital
Stewart, Mr. St. James's Square, Manchester
Swift, J. S. Esq. Surgeon, R. N.
Tedlie, Dr. Cape Coast Castle, Africa
Twemlow, Mr. Surgeon, Northwich
Warwick, Mr. Richard, Assistant Surgeon, R. N.
Wight, Dr. Robert, 15, Buccleugh Place, Edinburgh
Willis, Mr. Ratcliffe High Way
Valantine, Mr. J. W. Surgeon, Sutton-in-Ashfield, near Mansfield
Veitch, Dr. Grafton Street, London

### INDEX.

A

ABDOMINAL inflammation, cases of 35
Abdominal viscera, Dr. Percival on the 190
Abdomen, wound of the, in Algiers 464
Abercrombie, Dr. on the spinal cord 116
Abscesses in the liver 261
Absorption, M. Majendie on 431
Adams, Dr. his Life of John Hunter 430
Air pump vapour bath 203
Algiers, wounds received in the battle of 457
Amputation, the proper time for, after wounds 468
Analogy between hydrocephalus and scrofula 291
Anatomical examinations 429
Anatomy of hæmorrhoids 258
Aneurism of the heart, Dr. Carbutt on 118
Aneurism, case of 617
Aphthous ulcers 15
Armstrong, Dr. on scarlatina, &c. 46
——— on chronic diseases 55
——— on mania 197
——— on puerperal fever 583
Arnold, Dr. on cynanche laryngea 520
Arteria innominata, ligature of 263
Artery, subclavian, tied at Algiers 460
Ascites spontaneously cured 500
Asthma, Dr. Philip on 395
Astruc describes pseudo-syphilis 3
Atticus on fever 442
Ayre, Dr. on marasmus 399

B

Balfour, Dr. on emetic tartar 358
Barker, Dr. on fever 550
Bateman, Dr. on contagious fever 360
Belladonna, observations on 130
Bell's, Mr. Charles, quarterly report 209
Bell, Mr. Charles, remonstrated with 427
Bilious disorders, Dr. Ayre on 399
Blacklock, Mr. on hydrocephalus acutus 183
Bladder, a painful affection of the 181
Blaud, M. on compression of the carotids 498
Blood, transfusion of, by Dr. Blundel 521
Blood-letting in fever, Van Rotterdam on 71
BOOKS REVIEWED IN THE FIRST VOLUME. —— Doctor Armstrong on Scarlatina, &c. 46. Dr. Percival on Dublin Fevers, 63. Van Rotterdam on Blood-letting, 71. Dr. Robertson on Congestive Fever, 73. Dr. Williams on Gout, 77. Mr. Cooper on Exostosis, 79. Mr. Travers on Iritis, 87. Dr. Crampton on Periostitis, 95. Maclean on Epidemic and Pestilential Diseases, 187. Dr. Percival on Mania and Epilepsy, 190. Dr. Armstrong on Typhus, 197. Dr. Cheyne on Melena, 200. M. La Beaume on the Air-Pump Vapour Bath, 203 Dr. Scudamore on Gout and Rheumatism, 204. Sir Everard Home on the Prostate Gland, 206. Mr. Charles Bell's Surgical Observations, 209. Mr. Hunter on the Venereal Disease, 212. Mr. Todd on Hernia, 212. Mr. Dickenson on Burns and Scalds, 218. Mr. Young and Mr Thomas on Cancer, 219. Dr. Clarke's Report of the Lying-in Hospital, 220. Mr. Newnham on Inversio Uteri, 222. Dr. Wilson on Morbid Sympathies, 341. Dr. Reid on Tetanus and Hydrophobia, 353. Dr. Balfour on Emetic Tartar, 358. Dr. Bateman on Fever, 360 Mr. Mansford on Pulmonary Consumption, 375. Dr. Philip on the Vital Organs, 394. Dr. Ayre on Marasmus, 399. Mr. Carmichael on Venereal Diseases, 418. Orfila on Poisons, 505. Medico-Chirurgical Transactions, vol. ix. 516. Dr. Bradley on Stridor Abdominalis, 529. Dublin Hospital Reports, Vol. II. 541. Transactions of the King's and Queen's Colleges, Vol. II. 541. Dr. Hallaran on Insanity, 555. Mr. Brodie on Diseases of the Joints, 567. Dr. Armstrong on Puerperal Fever, 583.
Bostock, Dr. on paralysis 519
Botany, medical 601
Boyd, Dr. on tropical fever 115
Bradley, Dr. on stridor abdominalis 529

### INDEX.

Brien, Dr. on the evolution of the fœtus 123
Brodie, Mr. on diseases of the joints 567
Bronchotomy, Dr. Johnson on 119
Broussais on disordered circulation 103
——— his conversion in the treatment of fevers 451
Bruce, Mr. on the diseases at Marlow 526
Burder, Dr. on syphiloid diseases 1
Burgess and Hill's intelligencer 452
Burnett, Dr. case of gun-shot wound 28
Burns and scalds, Mr. Dickenson on 218
Bushel, Mr. on a preparation of kino 618

C

Cachexia syphiloidea 8
Cancer, Mr. Young on 219
Carbutt, Dr. on cardiac aneurism 118
——— medical cases 166, 476
Carmichael, Mr. on venereal diseases 418
——— in Reply to Mr. Guthrie 482
Carotids, compression of the 498
Cases of hydrocephalus 283
—— and dissections, Dr. Davies' 470
Catalogue Raisonnée 428, 601
Catarrhal disorders 132
Causes of syphiloid diseases 6
Cerebralis, Dr. on fever 437
Cheyne, Dr. on melena 200
———, Dr. on fever 542
Cholera morbus, pathology of 410, 417
Chronic ulcer with raised edges, syphiloid 19
——— diseases, Dr. Armstrong on 55
——— venous congestion 55
Clarke's, Dr. reports from the lying-in hospital 220
Cold water, accidents from drinking of, in heated states of the body 618
Concussion of the brain, Dr. Philip on 43
Congestive scarlatina 49
——— fever, Dr. Robertson on 73
Consumption, influenced by situation 375
Contagion, Dr. Percival on 63
Contagious fever, Dr. Bateman on 360
——— generated, *de novo* 361
Cookworthy, Dr. on extra-uterine conception 299
Cooper, Astley, on exostosis 79
Crampton, Dr. on periostitis 95
Cunningham, Mr. on injury of the head 122
Cynanche laryngea, Dr. Arnold on 520
——— tonsillaris, treatment of 618

D

Dangers of Dissection, M. Percy on the 233
Darwin, Dr. a successful practitioner, 344
Davis's, Dr. report on infantile diseases 448
———, Dr. report on diseases of children 249
———, Dr. remarkable cases 470
Dewar, Dr. on the wounded at Algiers 457
Diagnosis of syphilis and pseudo-syphilis 13
——— between Bulam and remittent fever 303
Dickenson, Mr. on burns and scalds 218
Dickson, Dr. on hydrencephalus 292
Dimsdale waters, Dr. Armstrong on the 59
Discussion on typhus, at Guy's Hospital 436
Dispensary, universal, for children 448
Dissection, dangers of 233
Dissertatio inauguralis, Dr. Burder's 1

E

Edmonston, Mr. on fever 113
Emetic tartar, Dr. Balfour on 358
Enteritis, Dr. Robertson on 150
Epidemic fevers of Dublin 63
Epilepsy, Maniacal 195
Erethism, mercurial, Dr. Bateman on 522
Erysipelas, cases of 615
Evans, Mr. on the Calcutta epidemic 156
Evolution of the fœtus, Dr. Brien on 123
Examination, anatomical 429
Excitement, Vialle on 103
Exostosis, Astley Cooper on 79
Extra-uterine conception, case of 299

F

Fauces, affections of the, in pseudo-syphilis 18
Felix, Dr. his case of obstinate plethora 25
Females, on the treatment of, by Dr. Moulson 31
Femoral artery, obliteration of 260
Fevers of Dublin, Dr. Percival on the 63
———, continued, Mr. Edmonston on 113
———, yellow, Mr. Niell on 303
——— anatomically investigated by Dr. Wight 311
——— is disturbance of all the functions 312

Fever, discussion on, at Guy's Hospital 436
——, yellow, Dr. Musgrave on 516
—— in Ireland, reports on 541
Forbes, Dr. on tropical fever 115
Frambesia, indigenous, in the West Indies 4
Functions, vital, Dr. Philip on 394

G

Galvanism in Asthma 395
Gastrotomy, case of 255
Gonorrhœa produces syphiloid diseases 11
Gout, Dr. Scudamore on 204
—— from sympathy with the digestive organs 348
Grabham's, Mr. case of monstrosity 617
Grant's observations on fevers 68
Grattan, Dr. on synocha and typhus 450
Gray's, Mr. supplement to the pharmacopœia, reviewed 428
Gregory, Dr. George, on dropsy 602
Guthrie, Mr. on phagedenic ulcer 323

H

Hæmorrhoids, anatomy of 258
——, Dr. Montegre on 137
Hallaran, Dr. on insanity 555
Hamilton, Mr. on laryngotomy 247
Harrowgate waters, Dr. Armstrong on the 59
Henderson's, Mr. dissections of hydrocephalus 289
Hepaticus, Dr. on fever 441
Hernia, Mr. Todd on 212
High operation for stone 451
Home, Sir E. on the prostate gland 206
Hospital reports, Dublin 95
—— pupil's guide 601
Hurtado, Dr. on abdominal inflammation 38
Hydrargyro-syphilitic iritis 91
Hydrencephalus, Dr. Yeats on 611
Hydrocephalic effusion incurable 291
Hydrocephalus acutus, Dr. Porter on 277
—— cases of 283
—— dissections of 284
——, Mr. Blacklock on 183
——, Dr. Johnson's case of 158
Hydrophobia, Dr. Reid on 353
——, Mr. Johnson on 494
Hysteria and hypochondriasis 259

I & J

Ichthyosis, case of, by Mr. Martin 521
Inequilibrium of circulation 103
Inflammation, abdominal, cases of 35
——, Dr. Porter's remarks on 281
Influence of situation in consumption 375
Ileus, case of 131
Intestines, wounds of 128
Insanity, Dr. Hallaran on 555
Inversio uteri, Mr. Newnham on 222
Iritis, Mr. Travers on 87
Jessore, sickness at 154
Johnson's, Dr. case of hydrocephalus 158
Johnson, Mr. D. on hydrophobia 494
Joints, Mr. Brodie on diseases of the 567

K

Kino, Mr. Bushel's preparation of 618

L

Laryngotomy, Mr. Hamilton on 247
Lectures on the eye, Mr. M'Kenzie's 430
Leveque's, Dr. cases of twins 41
Lithotomy above the pubes 451
Liver, abscesses in the 261
Lyon, Dr. on affections of the lungs 172

M

M'Kenzie's, Mr. William, lectures 430
M'Lean, Dr. his doctrines 361
—— on pestilential diseases 187
M'Whirter's, Dr. account of a surgical operation 614
Majendie, Dr. on absorption 431
Mania, Dr. Percival on 190
——, Dr. Armstrong on 197
Mansford, Mr. on situation in consumption 375
Manual of anatomy, Mr. Stanley's 429
Marasmus, Dr. Ayre on 399
Marlow College, report of diseases in 526
Medical pupilage, Dr. Johnson's 455
—— cases and remarks, Dr. Carbutt's 176, 476
Medico-Chirurgical Transactions 516
Medulla spinalis in fever 317
Melena, Dr. Cheyne on 200
Mills, Dr. on liver diseases 602
Monstrosity, Mr. Grabham's case of 617
Morbid anatomy, importance of 124
Moulson, Dr. on the treatment of females 31
——, Dr. on typhus 114
——, Dr. on paralysis 295
——, Dr. on cynanche tonsillaris 618
Musgrave, Dr. on yellow fever 216

N

Naval hospitals, syphilis in 1
Newnham, Mr. on inversio uteri 222

Niell, Mr. John, on yellow fever 303
Nomenclature, Dr. Porter's remarks on 277
Nux vomica in paralysis 254
———, Dr. Moulson on 297

O

O'Brien, Dr. on fever 541
Œsophagus, foreign bodies in the 261
Oil of turpentine, M. Recamier on, in sciatica 617
Operation, death from an 260
Oxalic acid 264

P

Paralysis cured by nux vomica 254
———, Dr. Moulson's cases of 295
Parturiology 220
Pathological observations 408
Percival, Dr. on fever 63
———, Dr. on the abdominal viscera 190
Periosteal exostosis 83
Periostitis, Dr. Crampton on 95
Periscope, quarterly 113, 237
Pestilential diseases, Dr. M'Lean on 187
Phagedenic ulcer 16, 19, 323
——— ulcer, Mr. Guthrie on the 323
——— ulcer, Mr. Carmichael on 423
Pharmacopœias, supplement to, Mr. Gray's 428
Philip, Dr. on concussion of the brain 43
———, Dr. on the vital functions 394
Phrenitis distinguished from hydrocephalus 280
Phthisis, pathology of 259
Plethora, obstinate case of 25
Poisons, epitome of 505
Pons varolii, its state in hydrocephalus 289
Porter, Dr. on hydrocephalus 277
———, Dr. on the epidemic fever 602
Portfolio 124, 436,
Power, loss of, over the voluntary muscles 519
Pregnancy, advanced states of 31
Preputial herpes 14
Primary syphiloid ulcers 14
Prostate gland, Sir E. Home on 206
Pseudo-syphilis, cases of 9
Puerperal fever, Dr. Armstrong on 583
Pupilage, medical, Dr. Johnson's 455

Q

Quarterly report in surgery, by C. Bell 209

R

Recamier, M. on oil of turpentine in sciatica 617
Registers, parish, Dr. Burrows on 430
Reid, Dr. on tetanus and hydrophobia 353
Resuscitation, case of 263
Rheumatism, acute, from morbid sympathy 347
Ribs, case of excision of 184
Richerand's case of excision of the ribs 184
Robertson, Dr. on congestive fever 73
———, Dr. on enteritis 150
———, Dr. Henry, on fever 115
Rostan, M. on aneurism 602

S

Sanders, Dr. on fever 319
Sanders, Dr. 292
Sarsaparilla useful in syphiloid diseases 23
Scarlet fever, Dr. Armstrong on 46
Scirrhus of the stomach, Mr. Bayle on 225
Scudamore, Dr. on gout 204
Secretions of females produce pseudo-syphilis 7
Semiology, observations on 608
Shearman's, Dr. report on infantile diseases 448
———, Dr. on diseases of children 249
Sheppard's, Mr. case of twins 39
Shoulder joint, amputation at the, by Dr. Burnett 28
Sight, case of loss of, by Mr. Williams 253
Silver, nitrate of, effects on the complexion 524
Situation, influence of, in consumption 375
Sivvens, a species of pseudo-syphilis 5
Sloughing ulcer 16
Somersetshire favourable to consumption 380
Spasmodic affections, Mr. Webster on 119
Spinal cord, Dr. Abercombie on the 116
Stanley's, Mr. manual of anatomy 429
Stilon, Mr. his case of visceral disease 273
Stoker, Dr. on fever 546
Stomach, scirrhus of the, M. Bayle on 225
———, fistulous wound of the 264
Stone, high operation for 451
Stridor abdominalis, Dr. Bradley on 529
Subclavian artery tied at Algiers 460
Subscribers, names of, 128, 272, 456, 620
Sulphuretted hydrogen gas in chronic diseases 59
Sulphur vapour baths 262
Superficial ulcers, syphiloid 15
Surgical operation, account of a 614

Sympathies, morbid, Dr. Wilson on 341
Syphilis 120
——— treated without mercury 324
———, true, less common than formerly 2
Syphiloid diseases, Dr. Burder on 1
——— diseases described by Celsus 2
——— diseases, causes of 6
——— buboes 17
——— symptoms, secondary 18
——— eruptions 20
——— affections of membranes and bones 21
——— diseases, treatment of 21

T

Taylor's, Dr. translation of Van Rotterdam 72
Tetanus, Dr. Reid on 353
Todd, Mr. on hernia 212
Tongue, state of the, in yellow fever 304
Toxicology, or epitome of poisons 505
Travers, Mr. on iritis 87
Tubercles, pseudo-syphilitic 15
Twins, cases of, by Mr. Sheppard 39
Typhus, Dr. Percival on 64
——— in Scotland 113
——— attended with trismus 114
———, simple, Dr. Bateman on 363
———, complicated, Dr. B. on 366
———, treatment of 369
———, discussion on, at Guy's Hospital 436
——— with pneumonia 476

U

Ulcers of the surface, syphiloid 15
Universal dispensary for children 240
Uterus, extirpation of the 618
———, case of chronic inversion of 618

V

Van Rotterdam on blood-letting 71
Vapour bath, M. La Beaume on 203
Vascularis [Dr. Armstrong] Dr. on fever 439
Venereal disease, Mr. Hunter on the 212
——— diseases, Mr. Carmichael on 418
Ventilation preferred to fumigation 372
Venus sine concubitu, Dr. Buchan's 601
Vialle on the circulation 103
Visceral disease, Mr. Stildn's case of 272
Vital functions, Dr. Philip on 394
Voluntary muscles, loss of power over 295
Vomiting, M. Chomel's case of 169

W

Webster, Mr. on influence of climate 115
Wight, Dr. on the nature of fever 311
Williams, Dr. on Wilson's tincture 77
———'s, Mr. case of loss of sight 253
Wilson's tincture, Dr. Williams on 77
———, Dr. on morbid sympathies 341
Wound, gun-shot, by Dr. Burnett 28
——— of the intestines 128
Wounds in the battle of Algiers 457
Wound of the thorax, severe 463

Y

Yeats, Dr. on hydrencephalus 611

Young, Mr. on cancer 219

———, Dr. N. L. on absorption 435

---

END OF VOL. I.

### TO THE PUBLIC.

The FIRST VOLUME of the Medico-Chirurgical Journal has closed, under an expression of public approbation, unprecedented in the Annals of Medical Literature. This approbation, while it affords the Editor an honourable source of independence, as well as gratification, insures a constant stimulus to increased exertion. A renovating health, a redoubling zeal, a spreading circle of correspondence, and a widening sphere of observation, shall all be combined—with indefatigable industry—to render this Journal worthy of the flattering patronage with which it has been already honoured by a generous and liberal public. To say more would be unnecessary—to say less would be ungrateful. It is by performances, not promises, that the Editor hopes to maintain the confidence of his professional brethren, and contribute his mite to the diffusion of Medical Science.

*Albany, Piccadilly, March* 25, 1819.

